# Ultra-fast real-time intraoperative diagnostic confirmation of successful sural nerve biopsy using digital confocal microscopy: a case report and review of the literature

**DOI:** 10.1136/bmjno-2024-000763

**Published:** 2025-06-15

**Authors:** James Selbie, William Bolton, Elizabeth Culpin, Ryan Mathew, Ian Anderson

**Affiliations:** 1 School of Medicine, University of Leeds, Leeds, UK; 2 Department of Neurosurgery, Leeds Teaching Hospitals NHS Trust, Leeds, UK; 3 University of Leeds, Leeds, UK; 4 Neurosurgery, Leeds General Infirmary, Leeds, UK

**Keywords:** NEUROSURGERY, PERIPHERAL NERVE SURGERY, PERIPHERAL NEUROPATHOLOGY

## Abstract

**Introduction:**

Sural nerve biopsy is a technique used to aid in the diagnosis of peripheral neuropathy. While this is considered a comparatively straightforward neurosurgical procedure, there is a recognised diagnostic ‘failure’ rate of around 4%, due to mistaken sectioning of the anatomically close and similar macroscopically appearing lesser saphenous vein.

**Case report:**

We report a case of suspected peripheral neuropathy, in which ultra-fast real-time digital confocal microscopy was used intraoperatively to rapidly confirm a successful sural nerve biopsy.

**Literature review:**

We conduct a literature review to determine current practice for the intraoperative confirmation of successful sural nerve biopsy.

**Discussion:**

Real-time confocal microscopy is used primarily for intraoperative tumour applications (in vivo margin assessment, ex vivo biopsy target confirmation). This is the first reported case of ultra-fast real-time digital confocal microscopy being used to confirm a successful sural nerve biopsy.

## Introduction

 Sural nerve biopsy is a technique used to establish the aetiology of peripheral neuropathy, especially when other diagnostic avenues have been exhausted. It is preferable to use whole biopsy over fascicular biopsy due to similar postoperative complication rates despite increased technical complexity and decreased diagnostic utility of the latter.^1^ Unfortunately, due to their close anatomical relationship and macroscopically similar appearances, it is common to mistakenly section the lesser saphenous vein instead of the sural nerve. This has been reported in up to 4% of cases.^2^ This is despite mitigation by checking the sample for fascicular tissue with the naked eye, or even under the operating microscope. The inevitable reoperation results in unnecessary discomfort for the patient and cost for the healthcare provider and potentially delays both diagnosis and treatment.

We report a case of a patient with clinically diagnosed peripheral neuropathy of unknown aetiology, in whom sural nerve biopsy was performed and diagnostic accuracy confirmed in theatre, using digital confocal microscopy.^3^ We also present a review of the literature to outline current practices for confirming successful nerve harvest, as well as illustrating how real-time histology technology can be used in this setting, to change practice for patient benefit.

## Case report

### History

A woman in her 30s presented with a 3-week history of ascending lower limb weakness and paraesthesia starting distally on a background of long-standing pain in the calves and feet bilaterally. She had no relevant medical history. She was initially investigated for spinal cord compression, with a spinal MRI, which was unremarkable. Electromyography was subsequently performed, and this showed severe axonal sensory and motor neuropathy affecting both lower limbs. A comprehensive blood panel, including immunological screen and testing for heavy metal toxicity, was found to be normal. A sural nerve biopsy was therefore undertaken.

### Surgery

The procedure was performed using the technique described in Hart *et al*,^4^ taking the ideal 4 cm of nerve. The patient felt pain on sharp cutting of the proximal end of the nerve. Both the operating microscope and a Histolog (SamanTree, Switzerland) machine were used to inspect the sample.

First, the biopsy was examined underneath the operating microscope, and a picture was taken (online supplemental figure 1). Although indistinct, fascicles could be seen. Together with the lack of clear lumen and glistening white cut surface, this suggested successful nerve biopsy.

Following this, a 1-centimetre piece was sharply cut from the biopsy for scanning using the Histolog. This involved dipping the sample in a fluorescent dye (The Histolog Dip, SamanTree, Switzerland) for 10 s, before it was rinsed and then placed on the flatbed scanner. The total time from sample preparation to the final image generation (online supplemental figure 2) was less than 1 min.

In contrast to the image obtained with the operating microscope, which could be described as suboptimal, the Histolog clearly demonstrated fascicular tissue, thus confirming that the sural nerve had been successfully biopsied.

Later, formal histopathological analysis (formalin-fixed paraffin-embedded sections, H&E) confirmed that the sample was indeed nervous tissue (online supplemental figure 3). We conclude that ultra-fast real-time digital confocal microscopy using the Histolog (SamanTree, Switzerland) may be relied on to confirm sural nerve biopsy intraoperatively at the time of surgical harvest.

## Discussion and literature review

### Current practices

#### Search

In order to find relevant articles, the PubMed database was searched using the terms (“sural nerve biopsy” OR “nerve biopsy” OR “nerve biopsies”) AND (“vein” OR “vessel”) OR “technique”. This yielded 127 results. Of these, papers that did not mention how to differentiate between nervous and venous tissue were excluded. On analysis of the remaining articles, three categories of technique were identified: clinical, visual inspection of the sample with the naked eye and intraoperative frozen section.

#### Results

One clinical indication of successful sural nerve harvest is a sharp, severe pain when the nerve is cut. However, this is not always the case.^4^

Permanent numbness in the area below the lateral malleolus almost always ensues after sural nerve biopsy. Therefore, this is a second clinical indication of successful harvest. However, this may not always be reliable due to the fact that patients with peripheral neuropathy often have such a significant disease that they are unable to detect any difference between baseline and postoperative sensation.^5^

Physical features of the sural nerve that can potentially be identified on visual inspection include the lack of a lumen, which would be seen in a vein,^6^ a glistening white appearance^7^ and acute-angle branches, which would not be seen in a vein.^8^

Chkheidze and Pytel^9^ describe how a frozen section can be ordered intraoperatively to conclusively determine whether nerve biopsy has been successful. This is a more reliable method than those described above but with the limitation that this adds potentially 30 to 45 min to the length of the procedure.^10^

### What is confocal microscopy?

Confocal microscopy is a technique which was first patented by Marvin Minsky in 1957.^11^ In contrast to conventional fluorescence microscopy, which illuminates an entire sample at once, confocal microscopy involves illumination and scanning of many discrete points within a sample. This enables the creation of a number of high-definition, cross-sectional images of the sample.^12^

The Histolog scanner, using this technique, was originally designed to facilitate intraoperative margin assessment of tumours.^3^ However, it can also be used to visualise other tissue types, such as peripheral nerves, as is evidenced by this case report.

## Conclusion

The Histolog (SamanTree, Switzerland) scanner enables rapid and risk-free confirmation of successful nerve harvest and does not require specialist staff to operate. The images acquired are of substantially higher resolution than those that can otherwise be obtained within the confines of the operating theatre. The sample is not damaged or consumed in any way and can undergo all standard downstream histopathological analyses. Therefore, although a 1-centimetre piece of nerve was cut from the 4 cm biopsy to undergo scanning, this does not represent the loss of diagnostic utility for any scanned tissue.

This technique is likely to reduce the risk of reoperation and allows the patient to be reassured that no further biopsies will be required.

There are no descriptions of using a Histolog machine for this purpose in the published literature, and we therefore believe this case to be a world first. There is no reason why this could not also be used to confirm the success of fascicular biopsies from other, more proximal nerves, at times when this is uncertain.

Further work could aim to determine whether histological images created intraoperatively using confocal microscopy are of sufficient quality to inform treatment, which would enable this to be started almost immediately.

## Supplementary material

10.1136/bmjno-2024-000763 online supplemental figure 1

## Data Availability

Data sharing is not applicable as no datasets were generated and/or analysed for this study.
