# Supplementary material for: Ultra-fast real-time intraoperative diagnostic confirmation of successful sural nerve biopsy using digital confocal microscopy: a case report and review of the literature
Source: BMJ Neurol Open. 2025 Jun 15;7(1):e000763. doi: 10.1136/bmjno-2024-000763 (PMC12314821; doi:10.1136/bmjno-2024-000763)
Supplement: online supplemental figure 1 [file bmjno-7-1-s001.pdf]

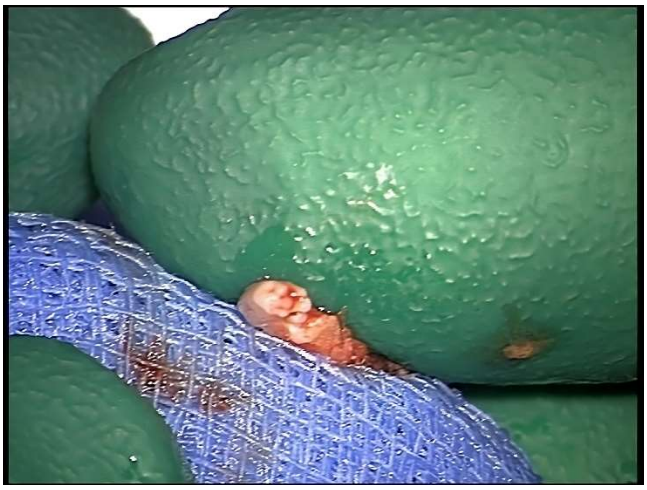

Supplemental figure 1: Operating microscope image of biopsy. Fascicles can be seen indistinctly.

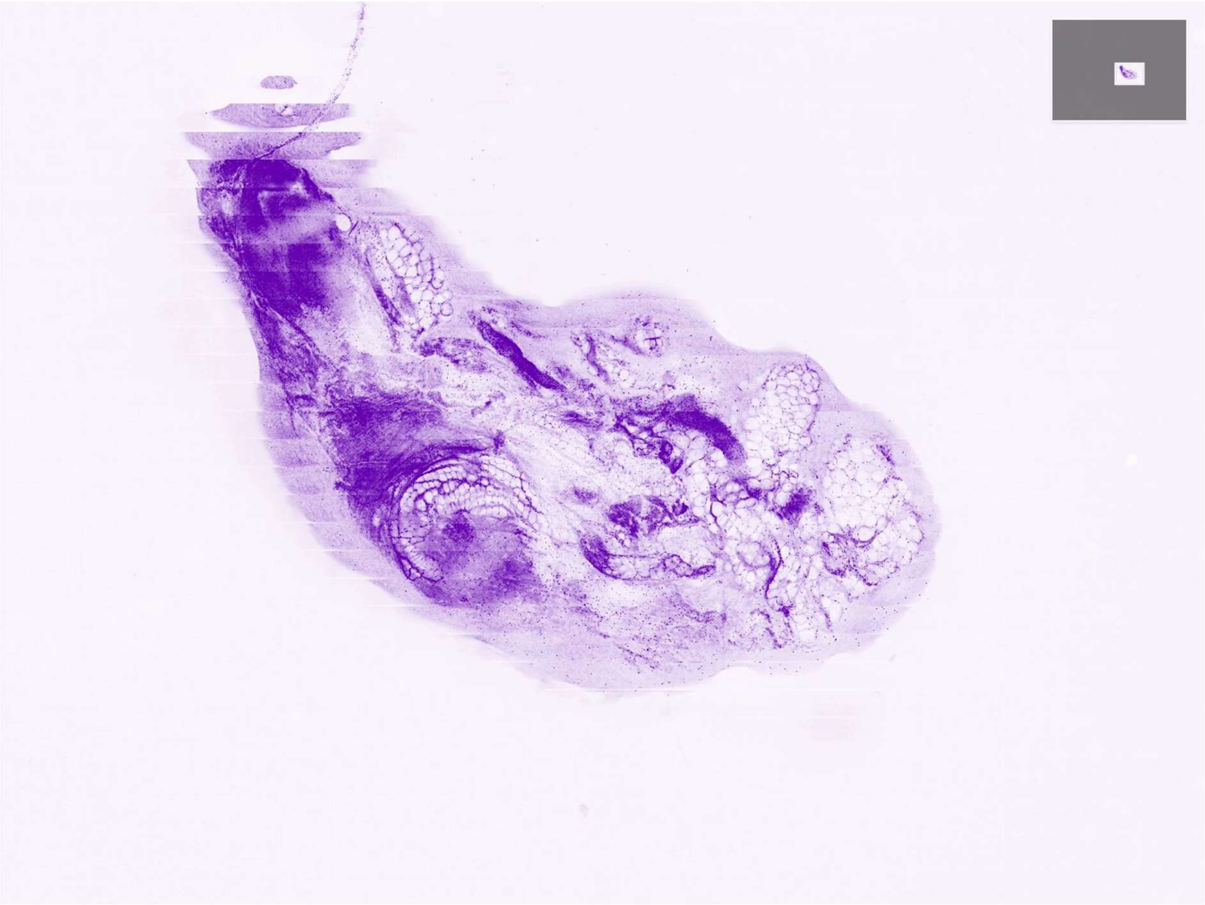

Supplemental figure 2: False H&E Histolog (SamanTree, Switzerland) digital confocal image of biopsy at 2 µm lateral resolution. Fascicles can be seen clearly.

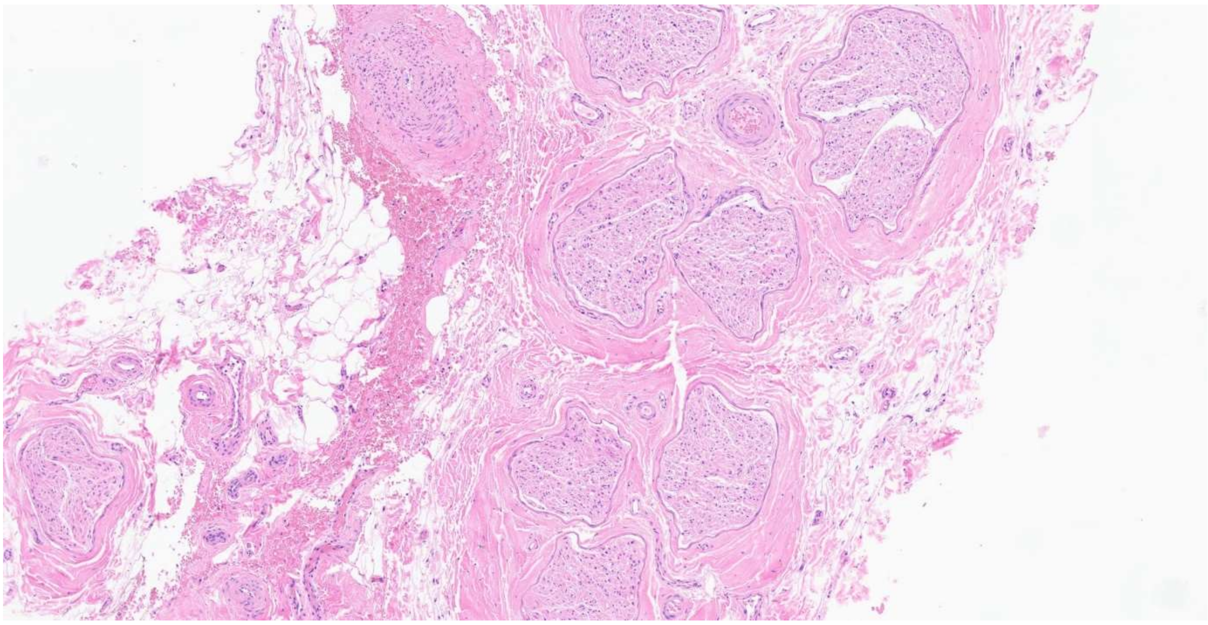

Supplemental figure 3: H&E stained section showing nerve fascicles.
